# Clinical and Pathological Characteristics of Metastatic Renal Cell Carcinoma Patients Needing a Second-Line Therapy: A Systematic Review

**DOI:** 10.3390/cancers12123634

**Published:** 2020-12-04

**Authors:** Nicola Longo, Marco Capece, Giuseppe Celentano, Roberto La Rocca, Gianluigi Califano, Claudia Collà Ruvolo, Carlo Buonerba, Fabio Esposito, Luigi Napolitano, Francesco Mangiapia, Ferdinando Fusco, Vincenzo Mirone, Massimiliano Creta

**Affiliations:** 1 Department of Neurosciences, Science of Reproduction and Odontostomatology, University of Naples Federico II, 80131 Naples, Italy; nicola.longo@unina.it (N.L.); marco.capece@unina.it (M.C.); roberto.larocca@unina.it (R.L.R.); gianl.califano2@gmail.com (G.C.); claudia.collaruvolo@unina.it (C.C.R.); fabio.esposito2@unina.it (F.E.); luigi.napolitano12@studenti.unina.it (L.N.); francesco.mangiapia@unina.it (F.M.); mirone@unina.it (V.M.); massimiliano.creta@unina.it (M.C.); 2 Department of Clinical Medicine and Surgery, University of Naples Federico II, 80131 Naples, Italy; carlo.buonerba@unina.it; 3 Department of Woman, Child and General and Specialized Surgery, Urology Unit, University of Campania ‘Luigi Vanvitelli’, 80131 Naples, Italy; ferdinando.fusco@unicampania.it

**Keywords:** metastatic, renal cell carcinoma, second line therapy

## Abstract

**Simple Summary:**

The management of metastatic renal cell carcinoma (mRCC) represents a clinical challenge. Progression or toxicity may occur during first-line treatments and many patients require a second-line option. Given the expanding options for second-line therapies clinicians are faced with the challenge to individualize treatment. We performed a systematic review in order to summarize available evidences about the clinicopathological profile of mRCC patients who receive a second-line therapy. We identified twenty-nine studies enrolling 7650 patients. Discontinuation of first-line therapy was due to progression in the majority of patients with 77.8% patients harboring ≥2 metastatic sites. Most patients had a good performance status, their age ranged from 55 to 70 years and their prognostic profile revealed a good or intermediate disease in most cases. Tailoring of second-line treatment strategies based on these features is strongly advocated.

**Abstract:**

A high percentage of patients with metastatic renal cell carcinoma (mRCC) require a second-line option. We aimed to summarize available evidences about the clinicopathological profile of mRCC patients who receive a second-line therapy. A systematic review was performed in August 2020. We included papers that met the following criteria: original research; English language; human studies; enrolling mRCC patients entering a second-line therapy. Twenty-nine studies enrolling 7650 patients (73.5% male, mean age: 55 to 70 years) were included. Clear cell histology was reported in 74.4% to 100% of cases. Tyrosine kinase inhibitors, immunotherapy, bevacizumab, mTOR inhibitors, and chemotherapy were adopted as first line option in 68.5%, 29.2%, 2.9%, 0.6%, and 0.2% of patients, respectively. Discontinuation of first-line therapy was due to progression and toxicity in 18.4% to 100% and in 17% to 48.8% of patients, respectively. Eastern Cooperative Oncology Group performance status score was 0 or 1 in most cases. Most prevalent prognostic categories according to the International Metastatic RCC Database Consortium and Memorial Sloan–Kettering Cancer Centre score were intermediate and good. About 77.8% of patients harboured ≥2 metastatic sites. In conclusion, patients who enter a second-line therapy are heterogeneous in terms of a clinical-pathological profile. Tailoring of second-line treatment strategies is strongly advocated.

## 1. Introduction

Renal cell carcinoma (RCC) accounts for about 3% of all cancers, with the highest incidence occurring in Western countries [1,2,3]. Approximately 25% of patients with RCC present with metastatic disease at diagnosis and up to 20% of those treated for early-stage disease will experience recurrence [1,2,3]. The overall incidence of metastatic RCC (mRCC) continues to rise by 2% per year. The landscape of therapy for patients with mRCC, has evolved dramatically over the past decade [2]. Prior to 2005, immunotherapy with interleukin-2 (IL-2) and interferon-α (IFN-α) represented the mainstay of therapy and median overall survival was about 1 year [2]. In 2005, the Food and Drug Administration approved sorafenib, the first vascular endothelial growth factor receptor (VEGFR)-targeted tyrosine kinase inhibitor (TKI) for RCC. The approval was closely followed by the introduction of several additional agents for advanced mRCC including other VEGFR-TKIs as well as mammalian target of rapamycin (mTOR) inhibitor therapies. These agents improved median survival estimates to approximately 2.5–3 years [2]. However, the management of mRCC still represents a clinical challenge [3]. Indeed, progression during first-line treatments may occur due to biological resistance mechanisms, and up to 60% of patients with mRCC require a second-line option with different mechanisms of action [3,4]. Moreover, treatment might be interrupted in some patients due to toxicity. In the second-line setting, treatment strategies have initially focused on vascular endothelial growth (VEGF) inhibition or switching toward inhibition of mechanistic target of mTOR [3]. Traditional second-line approaches include the mTOR inhibitor everolimus and axitinib, a selective VEGFR TKI [3]. Since 2015, three new second-line treatments have become available: cabozantinib, a TKI, nivolumab, an immuniocheckpoint inhibitor (ICI), and lenvatinib, a TKI used in combination with everolimus [3]. Given the expanding options for second-line therapies clinicians are facing with the challenge to individualize treatment [3]. Indeed, no conclusive data exist with respect to potential sequencing. The knowledge of demographic and clinical profile of patients with mRCC who enter a second-line therapy is considered of benefit for researchers involved in the identification of novel pharmacological strategies and for clinicians who are asked to personalize treatment strategies [5]. Currently, despite several evidences about molecular mechanisms involved in drug resistance to first-line therapy and clinical efficacy of second-line options in patients with mRCC, there are few evidences describing their demographic and clinical profile of mRCC patients who need a second-line regimen. The present systematic review aims to summarize available evidences about the clinicopathological profile of mRCC patients undergoing a second-line therapy.

## 2. Evidence Acquisition

This analysis was conducted and reported according to the general guidelines recommended by the Primary Reporting Items for Systematic Reviews and Meta-analyses (PRISMA) statement [6]. On August 2020 we performed a literature review to search for published studies demographic and clinical-pathological profile of mRCC patients who receive a second-line regimen. The search was performed in the Medline (US National Library of Medicine, Bethesda, MD, USA), Scopus (Elsevier, Amsterdam, The Netherlands) databases, and Web of Science Core Collection (Thomson Reuters, Toronto, ON, Canada). The following terms were combined to capture relevant publications: renal cell carcinoma (RCC), metastatic, resistant, toxicity, second line. We included full papers published in the last 15 years that met the following criteria: reporting original research; English language; human studies; enrolling mRCC patients who enter a second-line therapy. Reference lists in relevant articles and reviews were also screened for additional studies. Abstracts (with no subsequent full-text publications) and unpublished studies were not considered. Two authors (GC, CCR) reviewed the records separately and individually to select relevant publications, with any discrepancies resolved by a third author (NL). The following data were extracted from the studies included: first author, year of publication, enrollment period, sample size, ethnic origin, age, gender, tumor histology, tumor stage and grade, prior nephrectomy, first-line regimen, first-line progression free survival, first-line objective response rate, reasons for discontinuation, Eastern Cooperative Oncology Group performance status (ECOG PS) score, Memorial Sloan-Kettering Cancer Centre (MSKCC) score, International Metastatic Renal Cell Carcinoma Database Consortium (IMDC) score, number and site of metastatic sites, second line regimen. The quality of included studies was assessed using the Methodological Index for Non-Randomized Studies (MINORS) and the Jadad scores for non-randomized and randomized studies, respectively [7,8]. Ethical approval and patients’ consent were not required for the present study. 

## 3. Evidence Synthesis

**Figure 1:**
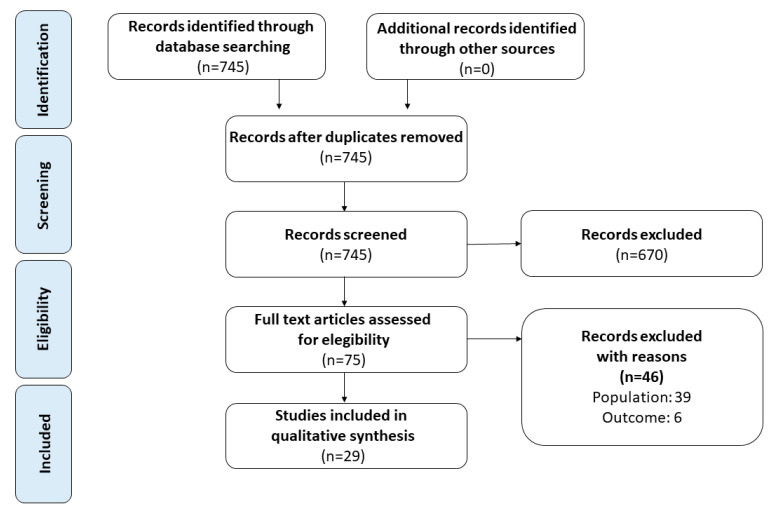
Flow diagram of the systematic review.

The search strategy revealed a total of 745 results. The screening of the titles and the abstracts defined 75 papers eligible for inclusion. Further assessment of eligibility, based on the study of the full-text papers, led to the exclusion of 46 papers. Twenty-nine studies were then included in the final analysis [9,10,11,12,13,14,15,16,17,18,19,20,21,22,23,24,25,26,27,28,29,30,31,32,33,34,35,36,37] (Figure 1).

Specifically, seven studies were randomized control trials (RCT) with Jadad score ranging from 1 to 5, six were prospective and 16 were retrospective, with methodological index for non-randomized studies (MINORS) score ranging from 8 to 18 (Table 1).

### 3.1. Patients’ Demographics and Tumor Characteristics

A total of 7650 patients who received second line therapy from 2003 to 2019 were included in the final analysis. Davis et al. [21] recorded the largest sample size (*n* = 1516), while Yoshida et al. [13] the smallest (*n* = 6). The patients’ demographics and characteristics of the tumour was not fully reported for all 7650 patients included and are summarized in Table 1. Mean age ranged from 55 to 70 years (range 19–89). Most of the patients included were white (*n* = 1671 out of 2143 reported (77.9%)), male (*n* = 5604, 73.5%) and with clear cell histology (ranged from 74.4% to 100%). Fuhrman or WHO/ISUP grade was only reported by one author [9]. Most patients (40.0% to 95.1%) had a ≥T3 stage disease. A total of 5371 (79.0% of 6793 reported) underwent nephrectomy. The percentage of patients who underwent prior nephrectomy ranged from 16.7% to 100%. Included studies failed to provide data about the type (radical vs. cytoreductive) and timing (upfront vs. delayed) of nephrectomy. 

### 3.2. Treatment History

Details about first-line treatment history are described in Table 2. 

#### 3.2.1. First Line Therapy

All the studies, except one [9], reported the first-line therapy drugs and the relative number of patients (*n* = 9027). Of those, 6187 patients (68.5%) received TKI (Sunitinib: 4528 (73.1%), Sorafenib: 880 (14.2%), Pazopanib: 637 (10.3%), Axitinib: 134 (2.1%), Tivozanib: 7 (0.1%), Cabozanitinib: 1 (0.01%)). Immunotherapy was administrated in 2637 (29.2%) patients (Interleukin and/or Interferon (1462, 55.4%), non-specified cytokine (1026, 38.9%), ICIs (149, 5.6%)). Moreover, 262 (2.9%) patients received Bevacizumab. In 59 patients (0.6%) mTOR inhibitors (Temsirolimus: 53 (89.8%), Everolimus: 6 (10.2%)) were administrated. Finally, 17 (0.2%) patients received chemotherapy (Thalidomide: 6 (35.3%), Lenalidomide: 5 (29.4%), Capecitabine: 3 (17.6%), Gemcitabine: 3 (17.6%)). 

#### 3.2.2. Progression Free Survival (PFS) and Objective Response Rates

Median PFS under first-line therapy was reported in 10 studies and ranged from 1.5 to 13.3 months. First-line response rate was reported in 5 studies (419 patients). In details, complete response (CR), partial response (PR), stable disease (SD) and progression disease (PD) was reported in 3 (0.7%), 94 (22.4%), 199 (47.5%) and 123 (29.3%) patients, respectively.

#### 3.2.3. Reason for Discontinuation

Reasons for discontinuation of first-line therapy were reported in 6 studies (262 patients). Specifically, 51 (19.4%) and 211 (80.6%) discontinued first line therapy because of toxicity and disease progression, respectively.

### 3.3. Disease Characteristics at Initiation of Second Line Therapy

Details about disease characteristics before starting second line therapy are described in Table 2. 

#### 3.3.1. Eastern Cooperative Oncology Group Performance Status (ECOG PS) Score

ECOG PS score at initiation of second-line therapy was reported in 15 studies enrolling a total of 4303 patients. Of those, 2092 patients (48.6%) showed an ECOG PS score of 0 whereas 2211 (52.4%) showed the ECOG PS score at progression ≥1.

#### 3.3.2. Prognostic Scores

Prognostic score before starting second-line therapy was reported in 23 studies enrolling 6583 patients. In details, MSKCC and the IMDC were used in 18 and 5 studies, respectively. The percentage of patients showing a favorable, intermediate, and poor prognostic score according to the MSKCC and IMDC scores were 31.8%, 53.9%, 14.3% and 8.7%, 65.3%, and 26%, respectively.

#### 3.3.3. Number of Metastasis and Metastatic Sites

The number of metastatic sites at progression was reported in 14 studies enrolling 1680 patients. One metastatic site was recorded in 372 (22.1%) patients. Conversely, in 1308 patients (77.8%) ≥2 sites were involved. Eighteen studies involving 4726 patients described the number of specific metastatic sites. The most frequent metastatic sites were lung, bones, lymph nodes and liver. Specifically, the number of patients harbouring lung, bone, lymph node and liver metastases were 1976 (41.8%), 763 (16.1%), 751 (15.9%) and 748 (15.8%), respectively. Less frequent metastatic sites were adrenal gland (*n* = 186, 3.9%), soft tissue (*n* = 133, 2.8%), central nervous system (*n* = 29, 0.6%), brain (*n* = 25, 0.5%), kidney (*n* = 12, 0.2%), mediastinum (*n* = 4, 0.1%) and chest wall (*n* = 1, 0.01%).

### 3.4. Second Line Therapy

Details about the type of second-line therapy were reported in 26 studies involving 5634 patients. Specifically, 2793 patients (49.6%) received mTOR inhibitors (Everolimus: 2107 (76.4%), Temsirolimus: 644 (23.0%), not specified: 42 (1.5%)). Tyrosin kinase inhibitors were administrated in 2170 patients (38.5%) (Axitinib: 739 (34.0%), Sunitinib: 664 (30.6%), Cabozanitinib: 423 (19.5%), Pazopanib: 193 (8.9%), Lenvatinib:110 (5.0%), not specified: 41 (1.9%)). Immunotherapy was given to 53 patients (0.9%) (Interleukin and/or Interferon (14, 26.4%), ICIs (39, 73.6%)). Moreover, 29 patients (0.5%) received Bevacizumab. Unspecified clinical trial drugs were administrated in 93 patients (1.6%) and 452 patients (8.0%) received placebo. Finally, 9 patients received Carfilzomib (0.1%) and 35 patients (0.6%) received Trebabanib. Detailed clinical and pathological prophile of mRCC patients according to second-line therapy was only possible in 18 studies [10,11,12,13,16,17,22,23,24,25,26,31,32,33,34,36,37]. Table 3 describes the available clinical and pathological features of mRCC patients stratified according the following second-line therapies: axitinib, cabozantinib, nivolumab, everolimus plus levatinib. The features of patients undergoing therapy with VEGF-targeted therapy in combination with immunotherapy could not be extracted. Mean age of patients when entering these second-line therapies was <65 years in all cases. The percentage of patients who underwent prior nephrectomy was lower among patients receiving axitinib (29.3%) and higher among those receiving nivolumab (87.2%). The percentage of patients with a good/intermediate prognostic profile was hugher among patients receiving cabozantinib (86.8%) and lower among those breceiving everolimus plus levatinib (60.4%).

## 4. Discussion

RCC incidence is rising at an average of 1.1% each year with 16% of the cases being metastatic at the time of presentation [5,38]. mRCC poses one of the great therapeutic challenges in oncology. Indeed, it is typically refractory to traditional cytotoxic chemotherapies, and until recently management options were limited to immunotherapy or palliative options. The paradigm of treatment and the prognosis of patients with mRCC has significantly changed in recent years thanks to the development and widespread use of molecular targeted agents, including VEGF pathway inhibitors, mammalian target of rapamycin pathway inhibitors, immune checkpoint inhibitors (ICIs). Since 2005, new first-line regimens have significantly improved the survival of mRCC patients. However, treatment discontinuation is often necessary due to disease progression, therapy-limiting toxicity, or patient request [9]. Thanks to recent improvements in targeted therapies clinicians have the opportunity to offer patients several lines of therapy. Nowadays, near half of patients with mRCC receive a second-line therapy [5]. The current European Association of Urology guidelines strongly recommend offering either nivolumab or cabozantinib for ICIs-naive VEGFr-refractory clear-cell mRCC and to offer any VEGF-targeted therapy that has not been previously used in combination with immunotherapy as second-line therapy for patients refractory to ICIs (strength of rating: weak) [39]. The National Comprehensive Cancer Network Guidelines recommend Cabozantinib, Nivolumab, Axitinib, and Lenvatinib plus Everolimus as category 1 after TKI treatment [40].

Nivolumab is an ICI antibody that disrupts the interaction of the PD-1 receptor with its ligands PD-L1 and PD-L2 [41]. It suppresses tumor growth by inducing the proliferation of cancer antigen-specific T cells and enhancing cytotoxic activity [41]. Axitinib is a potent, selective, second-generation inhibitor of vascular endothelial growth factor receptor (VEGFR)1, 2 and 3 [42]. Cabozantinib is a multitargeted receptor tyrosine kinase inhibitor with activity against hepatocyte growth factor receptor (tyrosine-protein kinase Met), vascular endothelial growth factor receptor 2 (VEGFR-2) and protoncogene tyrosine-protein kinase receptor Ret [43]. Lenvatinib is a small-molecule TKI that inhibits VEGFR1-3, fibroblast growth factor receptor (FGFR1-4), platelet-derived growth factor receptor α (PDGFRα), stem cell factor receptor (KIT), and rearranged during transfection (RET) [44]. Novel second-line treatment strategies have shown overall survival benefit up to 25 months compared to everolimus. However, the field of RCC treatments is evolving at a rapid and unprecedented pace that makes it difficult for researcher and clinicians to keep up with the latest evidence and derive the best recommendations and decisions. In the era of personalized medicine, we face the concrete difficulty of “targeting” available target therapies mainly due to the lack of reliable predictive factors, that are urgently needed. Beside molecular predictive factors, a detailed clinical-pathological picture of specific subsets of patients to treat is often required. Indeed, although guidelines are useful in the general population setting, clinicians are challenged with selecting treatments for individual patients. In this context, they have to consider a range of factors from the clinical-pathological profile, and prior therapy to less obvious but central issues in the daily life of patients [3]. To our knowledge, this is the first systematic review summarizing the demographic and clinicopathologic profile of mRCC patients who enter a second-line therapy. Our results provide the basis for many hypotheses that need to be tested in future investigations. Demographic features have relevant clinical implications for mRCC patients. Racial/ethnic and gender disparities have been described in terms of RCC incidence and survival. Black patients have been reported to have a significantly higher incidence rate and lower relative survival rate than all other races/ethnicities, whereas Asians/Pacific Islanders show an opposite trend [45]. 

A higher predominance in men over women has been described (1.5:1), together with a slightly lower relative survival rate [45]. Our results demonstrate that the majority of mRCC patients who receive a second-line therapy enrolled in clinical studies of captured in real-world databases have a Caucasian/White ethnic origin and are male. The relevance of ethnicity in terms of mRCC response to first-line therapies is widely under-investigated and deserves future evaluations. Rose et al. demonstrated that both Caucasian and African American patients with mRCC had a significant increase in rates of systemic treatment with an accompanying improvement in survival since the introduction of targeted therapies [46]. However, African American patients showed a survival disadvantage compared to Caucasians independent of treatment received, probably due to tumour biology, comorbidities, or disease burden [46]. The authors hypothesized that the racial disparity in survival may be related to factors unaffected by the implementation of therapies and that treatment bias does not explain the survival disparity [46]. Although gene polymorphism may explain the disparity of response and tolerability in mRCC patients receiving targeted therapy, further studies about the exact mechanism are required. Interestingly, the male to female ratio we observed when describing the population of mRCC is higher than the 1.5:1 incidence ratio. This finding leads to hypothesize a gender difference in terms of tumor progression and/or drug toxicity. Gender influences epidemiology, histology, surgical treatment, complications, response to medical therapy, and long-term oncological and functional outcomes in RCC [47,48]. In detail, the male gender has been associated with worse RCC clinical features and prognosis. The reason of such discrepancy should be further evaluated, as it could be related either to the immune-related genes of the X chromosome or to the hormonal sex influences on cancer susceptibility or both [47]. Furthermore, a gender selection bias should also be considered as a potential explanation for this observation. Indeed, as recently reported by Mancini et al., men are included in clinical trials and prospective studies on genitourinary cancers more often than women [48,49]. A better clarification of gender-related mechanisms can lead to the possibility of including gender factors in risk-predictive nomograms and allow the possibility for personalized gender-oriented treatment options [48,49].

Mean patient ages range from 57 to 70 years. Currently, uncertainties exist about the prognostic effect of age on RCC. Some authors have pointed out that older age is correlated with a higher stage and pathological grade, suggesting an adverse association with prognosis [50]. In their study, Zhang et al. found that younger patients with mRCC receiving targeted therapy had a poorer prognosis compared with older patients [50]. Interestingly, the mean age of patients receiving axitinib, cabozantinib, nivolumab, and the combination of everolimus plus levatinib was < 65 years. Of note, younger patients also have theoretically a low comorbidity status and can better tolerate further lines of treatment. The age profile emphasizes the need to improve the accessibility to second lines of treatment. Moreover, this evidence points out the need for further studies assessing the outcomes of second-line therapies in older patients. 

Each kidney cancer histology has unique genomic and clinical features that should be taken into account when planning appropriate targeted therapies [51]. Clear cell RCC represents approximately 75% of renal cancers. As expected, clear-cell histology is highly prevalent among the mRCC population captured by our review. However, non-clear cell histology is reported in up to 25.6% of these patients. Unfortunately, non-clear cell kidney cancer still represents an unmet need from a therapeutic point of view and available treatments have demonstrated limited efficacy in this subset of patients [51].

The majority of patients entering a second-line therapy discontinued first-line drugs due to disease progression. However, a non-negligible percentage of them (up to 48.8%) discontinued it due to toxicity. This finding has relevant clinical implications. Although demonstrated only for mRCC patients who discontinue VEGF-targeted therapies, it has been reported that patients who discontinue first-line therapy because of toxicity have better outcomes than patients who stop it because of disease progression [51]. Whether the former subset of patients should receive different consideration when starting next line of therapy still remains a controversial issue [52].

The number and typology of metastatic sites have a relevant prognostic role in mRCC patients. Patients with only one metastatic site have been reported to have a better prognosis when compared to patients with multiple sites involved [53]. Although most patients entering a second line therapy have more than one metastatic site, there is a considerable percentage of patients with only one site involved. Several authors demonstrated variable outcomes depending on the patterns of metastasis. Although the lung is the most frequently involved metastatic site in patients undergoing a second-line therapy, our analysis points out a heterogeneous distribution of metastatic sites. Typically, bone and brain metastases represent significant therapeutic dilemmas as they are poorly responsive to medical therapy [2]. Bone is involved in a significant number of mRCC patients entering a second-line therapy. Published data have pointed to the potential utility of cabozantinib in patients with bone metastasis, thus providing a potential rationale to personalize second-line therapies according to the metastatic sites [2].

Although most mRCC patients receiving second-line therapy had a prior nephrectomy, a significant percentage of them (up to 83.3% in some series) did not receive surgery. The role of cytoreductive nephrectomy (CN) has profoundly changed in recent years along with the evolution of medical therapy [53]. The theoretical benefits of CN include facilitation of spontaneous regression, reduction of de novo metastases, and palliation of symptoms [54]. However, these potential benefits must be considered in the context of perioperative morbidity and the delayed receipt of systemic treatments. In the cytokine era, CN provided a crystal-clear benefit in terms of overall survival and it was considered the standard of care [54]. More recently, based on the results of the CARMENA and SURTIME trials, patients with MSKCC intermediate- and poor-risk are deemed not suitable for upfront CN as this will delay the beginning of target therapy thus potentially decreasing the overall survival [54]. Therefore, although CN still remains an important tool in the multimodality management of mRCC, careful patient selection is of paramount importance and discussion in multidisciplinary teams is required. To date, the role of CN in the setting of ICI remains largely undefined and future trials are required to provide insight on patient selection and optimal timing of CN in this clinical scenario [54]. Stratification of mRCC according to prognostic models has relevant clinical implications and guidelines recommend tailoring first-line therapies accordingly. Most patients receiving a second-line therapy belong to the favorable/intermediate prognostic categories with the latter being the most represented in most series. Future investigations are required to explore the role of second-line agents’ selection according to the prognostic risk category. 

The potential limitations of this review must be acknowledged: available studies often provide incomplete and heterogeneously reported clinicopathologic data. In most cases, patients enrolled in the included studies are selected on the basis of predefined inclusion and exclusion criteria thus being not completely representative of patients found in everyday clinical practice. Finally, this study simply describes the characteristics of patients who receive a second-line regimen while future studies are needed to depict the profile of the entire population of patients who discontinue a first line regimen. 

## 5. Conclusions

Based on data from both clinical trials and real-life observational registries, patients who are submitted to second-line therapy represent a heterogeneous group. Most of the reported cases, however, show a good performance status, are younger than 70 years and have a good/intermediate prognostic profile. Future studies are needed to better characterize profiles and subtypes of patients submitted to second-line treatments.

## Figures and Tables

**Table 1:** Patients’ demographics and tumor characteristics.

Author (Year)	Study Design	Study Period	Jadad Score	MINOR Score	Sample Size (*n*)	Ethnic Origin (*n*)	Age at Progression Mean (Range)	Male:Female	Histology of Primary Tumour (%)	T-Stage (%)	Fuhrman or WHO/ISUP Grade (%)	Nephrectomy *n* (%)
Suzuki (2020) (a) [10]	R	2016–2019	-	14	41	n/a	70 (46–88)	33:8	Clear Cell (82.9) Other (17.1)	n/a	n/a	34 (82.9)
Suzuki (2020) (b) [10]					39		67 (39–87)	29:10	Clear Cell (74.4) Other (25.6)			34 (87.2)
Tomita (2020) [11]	P	2017–2020	-	-	35	n/a	63 (42–84)	24:11	Clear Cell (100)	n/a	n/a	34 (97.1)
Hamieh (2020) [12]	P	n/a–2019	-	-	7	Caucasian (7)	57 (39–63)	7:0	Clear Cell (100)	n/a	n/a	6 (86.0)
Yoshida (2019) [13]	R	n/a–2018	-	8	6	n/a	65.2 (49–83)	5:1	Clear Cell (83.3) Acquried cystic disease associated RCC (16.7)	T1b (16.6) T2 (16.6) T3a (66.6)	n/a	6 (100)
Shah (2019) [14]	R	2015–2018		11	70	n/a	59 (44–75)	50:20	Clear Cell (100)	n/a	n/a	60 (86.0)
Bersanelli (2019) [15]	R	2005–2011	-	12	150	n/a	n/a	115:35	Clear Cell (77.0) Papillary (13.5) Pure sarcomatoid (5.4) Sarcomatoid component (13.0) Others (4.0)	T1 (6.0) T2 (14.0) T3 (58.0) T4 (8.7)	n/a	129 (86.0)
Hasanov (2019) [16]	P	2013–2019	-	-	9	White or Caucasian (8) Hispanic or Latino (1)	59 (53–73)	5:4	Clear Cell (100)	n/a	n/a	8 (89.0)
Semrad (2018) (a) [17]	RCT	2012–2018	3	-	17	White (9) American Indian/Alaska native (2) Black (2) Hispanic (4)	64 (49–76)	13:4	Clear Cell (100)	n/a	n/a	n/a
Semrad (2018) (b) [17]					18	White (12) Asian/Pacific Islander (1) Black (1) Hispanic (4)	59 (46–74)	14:4				
Auvray (2018) [18]	R	2015–2018		12	33	n/a	61 (40–77)	23:10	Clear Cell (100)	n/a	n	25 (76.0)
Ishihara (2017) [19]	R	2007–2016	-	9	60	n/a	n/a	42:18	Clear Cell (76.7) Other (23.3)	n/a	n/a	n/a
Lakomy (2017) [20]	R	2014–2016	-	13	1029	n/a	59 (33–81)	740:248	Clear Cell (94.1) Papillary (4.85) Other (1.05)	n/a	n/a	849 (85.9)
Eggers (2017) [9]	R	2005–2012	-	10	105	n/a	n/a	74:31	Clear Cell (83.2) Papillary (4.3) Other (4.4)	T1 (15.2) T2 (21.0) T3 (40.0) T4 (3.8)	G1 (8.6) G2/3: (80.9)	n/a
Davis (2016) [21]	R	2003–2015		10	1516	n/a	n/a	1110:406	Clear Cell (89.0) Other (11.0) Sarcomatoid component (11.0)	n/a	n/a	1256 (83.0)
D’Aniello (2016) [22]	R	2014–2016	-	8	62	n/a	62 (36–86) *	55:7	Clear Cell (94.2) Other (4.8)	n/a	n/a	54 (87.1)
Motzer (2015) (a) [23]	RCT	2012–2013	4	-	51	n/a	61 (44–79)	35:16	Clear Cell (100)	n/a	n/a	44 (86.0)
Motzer (2015) (b) [23]					52		64 (41–79)	39:13				43 (83.0)
Motzer (2015) (c) [23]					50		59 (37–77)	38:12				48 (96.0)
Choueiri (2015) (a) [24]	RCT	2013–2014	3	-	330	White (269) Asian (21) Black (6) Other (19) Not reported (15) Missing (0)	63 (32–86)	253:77	n/a	n/a	n/a	284 (86.0)
Choueiri (2015) (b) [24]					328	White (263) Asian (26) Black (3) Other (13) Not reported (22) Missing (1)	62 (31–84)	241:86				280 (85.0)
Bergmann (2015) [25]	P	2009–2013	-	-	334	n/a	68 (22–89)	250:84	Clear Cell (88.0) Non-Clear Cell (7.0) Missing (5.0)	n/a	n/a	300 (90.0)
Hutson (2014) (a) [26]	RCT	2007–2011	4	-	259	White (178) Asian (38) Other (43)	60 (19–82)	193:66	Clear Cell (83.0) Non-Clear Cell (17.0)	n/a	n/a	223 (86.0)
Hutson (2014) (b) [26]					253	White (163) Asian (50) Other (40)	61 (21–80)	192:61	Clear Cell (82.0) Non-Clear Cell (18.0)			219 (87.0)
Signorovitch (2014) [27]	R	2019–2012	-	12	281	n/a	n/a	182:99	Clear Cell (84.0) Non-Clear Cell (16.0)	n/a	n/a	130 (46.3)
Wong (2014) [28]	R	2011	-	13	534	White (421) Others (113)	64 (34–88)	376:158	Clear Cell (89.0) Non-Clear Cell (11.0)	n/a	n/a	89 (16.7)
Park (2012) [29]	R	2005–2011		14	83	n/a	55 (26–84)	61:22	Clear Cell (78.0) Non-Clear Cell (22.0)	n/a	n/a	67 (81.0)
Busch (2013) [30]	R	2005–2011	-	18	103	n/a	n/a	67:36	Clear Cell (86.0) Non-Clear Cell (10.0) Unknown (7.0)	n/a	n/a	100 (97.0)
Trask (2011) [31]	RCT	2006	1	-	62	White (60) Asian (1) Other (1)	n/a	42:20	Clear Cell (82.2) Other (17.8)	T4 (95.1) Other (4.9)	n/a	62 (100)
Rini (2011) (a) [32]	RCT	2008–2010	4	-	361	White (278) Black (1) Asian (77) Other (5)	61 (20–82)	265:96	Clear Cell (100)	n/a	n/a	n/a
Rini (2011) (b) [32]					362	White (278) Black (1) Asian (77) Other (5)	61 (22–80)	258:104				
Zimmerman (2009) [33]	R	2005–2006	-	12	22	n/a	61 (39–78)	16:6	Clear Cell (100)	n/a	n/a	12 (54.5)
Di Lorenzo (2009) [34]	P	2006–2008	-	-	52	n/a	60 (40–78)	35:17	Clear Cell (86.5) Papillary (9.6) Sarcomatoid (3.8)	n/a	n/a	49 (94.2)
Tamaskar (2008) [35]	R	n/a	-	12	30	n/a	62 (42–77)	24:6	Clear Cell (93.3) Papillary + Clear Cell (6.6)	n/a	n/a	30 (100)
Motzer (2006) [36]	P	2003	-	-	63	n/a	60 (24–87)	43:20	Clear Cell (87.0) Papillary (6.0) Sarcomatoid variant (2.0) Unknown (5.0)	n/a	n/a	58 (92.0)
Escudier (2004) (a) [37]	RCT	2003–2005	5	-	451	n/a	58 (19–86)	315:58	Clear Cell (100)	n/a	n/a	422 (94.0)
Escudier (2004) (b) [37]					452		59 (29–84)	340:59	Clear Cell (100)			421 (93.0)

MINORS: Methodological Index for Non-Randomized Studies; P: prospective; R: retrospective, RCT: randomized controlled trial; RCC: renal cell carcinoma; n/a: not available; *: median (IQR).

**Table 2:** Treatment history and disease characteristics at initiation of second line therapy.

Author (Year)	First Line Regimen (*n*)	Reason for Discontinuation *n* (%)		First-line PFS (Months) Mean (Range)	First-Line Response Rate (%)	ECOG PS Score (*n*)	Prognostic Category *n* (%)			Metastatic Sites (*n*)	Involved Metastatic Sites (*n*)	Second Line Regimen (%)
		Toxicity	Progression				Favorable /Good	Intermediate	Poor			
Suzuki (2020) (a) [10]	Sunitinib (18) Pazopanib (19) Sorafenib (2) Temsirolimus (2)	20 (48.8)	21 (51.2)	12.7(6.2–45.1)	n/a	n/a	3 (7.3) ^#^	24 (58.5) ^#^	14 (34.2) ^#^	1 (23) ≥2 (18)	n/a	Axitinib 41 (100)
Suzuki (2020) (b) [10]	Sunitinib (20) Pazopanib (18) Sorafenib (1)	11 (28.2)	28 (71.8)	13.3 (7.1 -16.9)	n/a		2 (25.1) ^#^	23 (59.0) ^#^	14 (35.9) ^#^	1 (21) ≥2 (18)		Nivolumab 39 (100)
Tomita (2020) [11]	Sunitinib (24) Axitinib (18) Pazopanib (7) Nivolumab (11) Avelumab (3) Pembrolizumab (1)	n/a	n/a	n/a	n/a	n/a	11 (31.4) °	19 (62.9) °	5 (14.3)	1 (6) 2 (11) ≥3 (3)	Bone (8) Lung (21) Liver (9) Lung or liver, and bone (25) Lymph node (11) Other (15)	Cabozantinib 35 (100)
Hamieh (2020) [12]	Sunitinib (2) Pazopanib (1) Ipilimumab + Nivolumab (3) Cabozantinib (1)	n/a	n/a	1.5 (0.8 -3.0)	n/a	n/a	0 (0) ^#^	4 (57.1) ^#^	3 (42.8) ^#^	n/a	Lung (6) Bone (3) Brain (4) Liver (1)	Lenvatinib + Everolisimus 7 (100)
Yoshida (2019) [13]	Sorafenib (2) Sunitinib (3) IL2 (1) + Nivolumab	n/a	n/a	n/a	n/a	n/a	0 (0) ^#^	6 (100) ^#^	0 (0) ^#^	1 (2) 2 (3) 3 (1)	Lung (n/a) Lymph node (n/a) Right adrenal gland (n/a)	Axitinib 6 (100)
Shah (2019) [14]	Anti-PD-(L)1 single agent (12) PD-1 + CTLA-4 blockade (33) PD-(L)1 + anti-VEGF therapy (25)	12 (17.0)	58 (83.0)	n/a	n/a	n/a	8 (11.0) ^#^	48 (69.0) ^#^	14 (20.0) ^#^	n/a	Lung (61) Bone (35) Liver (12) Lymph node (48) Adrenal gland (22)	Pazopanib 19 (27) Sunitinib 6 (9) Axitinib 25 (36) Cabozantinib 20 (28)
Bersanelli (2019) [15]	Sunitinib (150)	n/a (26.3)	n/a (61.7)	n/a	n/a	n/a	16 (10.7) °	95 (63.7) °	28 (18.9) °	1 (19) 2 (33) ≥3 (48)	Lung (70) Lymph node (59) Bone (31) Liver (25) Brain (11) Renal bed (9)	VEGF -TKI (n/a) mTORI (n/a)
Hasanov (2019) [16]	Sunitinib (7) Everolimus (6) Pazopanib (6) Temsirolimus (4) Capecitabine (3) Gemcitabine (3) Axitinib (2) Bevacizumab (1) Sorafenib (1) Tivozanib (1)	n/a	n/a	1.8 (0.8–3.6)	n/a	0 (6) 1 (2) 2 (1)	1 (11.0) °	6 (67) °	2 (22) °	1 (1) 2 (3) 3 (1) 4 (2) 6 (1) 10 (1)	Lung (8) Mediastinum (4) Liver (3) Lymph node (2) Chest wall (1)	Carfilzomib 9 (100)
Semrad (2018) (a) [17]	Bevacizumab (5) Pazopanib (6) Sorafenib (2) Sunitinib (4)	n/a	n/a	n/a	n/a	0 (12) 1 (5)	n/a			n/a	n/a	Trebabanib 17 (48.5)
Semrad (2018) (b) [17]	Bevacizumab (10) Pazopanib (5) Sorafenib (2) Sunitinib (1)					0 11) 1 (7)						Trebabanib + anti VEGF 18 (51.5)
Auvray (2018) [18]	Nivolumab -ipilimumab (33)	8 (24.2)	25 (75.8)	8.0 (5.0–13.0)	n/a	n/a	4 (12.1) ^#^	23 (69.7) ^#^	6 (18.2) ^#^	n/a	n/a	Sunitinib 17 (51.5) Axitinib 8 (24.2) Pazopanib 6 (18.2) Cabozantinib 2 (6.1)
Ishihara (2017) [19]	Sunitinib (37) Sorafenib (21) Pazopanib (2)	0 (0)	60 (100)	n/a	n/a	n/a	9 (15.0) °	44 (73.3) °	7 (11.7) °	1 (18) ≥2 (42)	Lung (50) Liver (10) Bone (12) Lymph node (19)	Sunitinib13 (21.6) Sorafenib 2 (3.69) Axitinib 30 (50) Pazopanib 3 (5) Temsirolimus 4 (6.7) Everolimus 8 (13.3)
Lakomy (2017) [20]	Bevacizumab + interferon-alpha (35) Sorafenib (232) Sunitinib (655) Temsirolimus (23) Pazopanib (84)	n/a	n/a	10 (n/a)	n/a	0 (182) 1 (487) 2 (46) 3 (1) Unknown (272)	361 (36.5) °	573 (58.0) °	54 (5.46) °	n/a	n/a	Everolimus 520 (50.5) Sorafenib 240 (23.3) Sunitinib 228 (22.1) Axitinib 29 (2.8) Pazopanib 10 (0.97) Temsirolimus 1 (0.09) Bevacizumab + interferon-alpha 1 (0.09)
Eggers (2017) [9]	Sunitinib (n/a) Sorafenib (n/a) Axitinib (n/a) Pazopanib (n/a) Cytokine (n/a)	n/a	n/a	n/a	n/a	0 (75) ≥1 (8) n/a (22)	8 (7.6) °	30 (28.6) °	2 (1.9) °	1 (44) >1 (41) n/a (20)	n/a	n/a
Davis (2016) [21]	Sunitinib (1068) Sorafenib (279) Axitinib (4) Bevacizumab (55) Pazopanib (110)	n/a	n/a	8.1 (3.9-16.0)	n/a	n/a	329 (22) °	902 (60) °	285 (19) °	n/a	n/a	Sunitinib 278 (18.0) Sorafenib 325 (21.0) Axitinib 107 (7.1) Pazopanib 120 (7.9) Cabozantinib 16 (1.1) Bevacizumab 28 (1.8) Temsirolimus 133 (8.8) Everolimus 403 (27.0) INF/IL-2 13 (0.9) Clinical trial drugs 93 (6.1)
D’Aniello (2016) [22]	Sunitinib (62)	n/a	n/a	7.18 (4.04-13.4)	n/a	0 (42) 1 (18) 2 (2)	15 (24.2) °	43 (69.4) °	4 (6.5) °	n/a	Lung: (29) Bone: (8) Liver: (4) Lymph-node: (9) Other: (12)	Axitinib 62 (100)
Motzer (2015) (a) [23]	Axitinib (1) Bevacizumab (0) Pazopanib (9) Sorafenib (1) Sunitinib (36) Tivozanib (3) Other (1)	n/a	n/a	n/a	CR 1 (2) PR 14 (28) SD 20 (39) PD 7 (14) n/a 9 (18)	0 (27) 1 (24)	12 (24.0) °	19 (37.0) °	20 (39.0) °	1 (18) 2 (15) ≥3 (18)	Bone (12) Liver (10) Lung (27) Lymph nodes (25)	Lenvatinib + Everolimus 51 (100)
Motzer (2015) (b) [23]	Axitinib (2) Bevacizumab (1) Pazopanib (13) Sorafenib (0) Sunitinib (35) Tivozanib (1) Other (0)				PR 10 (19) SD 28 (54) PD 10 (19) n/a 4 (8)	0 (29) 1 (23)	11 (21.0) °	18 (35.0) °	23 (44.0) °	1 (9) 2 (15) ≥3 (28)	Bone (13) Liver (14) Lung (35) Lymph nodes (31)	Single agent Lenvatinib 52 (100)
Motzer (2015) (c) [23]	Axitinib (0) Bevacizumab (4) Pazopanib (13) Sorafenib (2) Sunitinib (28) Tivozanib (2) Other (1)				PR 10 (20) SD 21 (42) PD 15 (30) n/a 9 (8)	0 (28) 1 (22)	12 (24.0) °	19 (38.0) °	19 (38.0) °	1 (5) 2 (15) ≥3 (30)	Bone (16) Liver (13) Lung (35) Lymph nodes (33)	Single agent Everolimus 50 (100)
Choueiri (2015) (a) [24]	Sunitinib (210) Pazopanib (144) Axitinib (52) Sorafenib (21) Bevacizumab (5) IL-2 (20) Interferon alfa (19) Nivolumab (17)	n/a	n/a	n/a	n/a	0 (226) 1 (104)	150 (45.0) °	137 (42.0) °	43 (13.0) °	n/a	n/a	Cabozantinib 330 (50.1)
Choueiri (2015) (b) [24]	Sunitinib (205) Pazopanib (136) Axitinib (55) Sorafenib (31) Bevacizumab (11) IL-2 (29) Interferon alfa (24) Nivolumab (14)					0 (217) 1 (111)	150 (46.0) °	135 (41.0) °	43 (13.0) °			Everolimus 328 (49.9)
Bergmann (2015) [25]	Sunitinib (260) Sorafenib (68) Pazopanib (12) Bevacizumab (41) Cytokines (33)	n/a	n/a	n/a	n/a	n/a	84 (35.0) °	134 (56.0) °	20 (8.0) °	n/a	Lung (226) Lymph node (145) Bone (125) Liver (87) Adrenal gland (47)	Everolimus 334 (100)
Hutson (2014) (a) [26]	Sunitinib (259)	n/a	n/a	n/a	n/a	0 (103) 1 (150) Other (6)	50 (19.0) °	178 (69.0) °	31 (12.0) °	n/a	n/a	Temsirolsimus 259 (100)
Hutson (2014) (b) [26]	Sunitinib (253)					0 (113) 1 (139) Other (1)	44 (17.0) °	177 (70.0) °	32 (13.0) °			Sorafenib 253 (100)
Signorovitch (2014) [27]	Sunitinib (206) Sorafenib (49) Pazopanib (26)	n/a	n/a	n/a	n/a	0 (40) ≥1 (234)	67 (23.8) °	138 (49.1) °	30 (10.7) °	n/a	Lung (232) Lymph nodes (152) Bone (148) Liver (76) Adrenal gland (35) Soft tissue (49) Central nervous system (13) Other (6)	Everolimus 138 (49.1) Temsirolimus 64 (22.8) Sorafenib 20 (7.1) Sunitinib 16 (5.7) Pazopanib 35 (12.5) Axitinib 8 (2.8)
Wong (2014) [28]	Sunitinib (459) Sorafenib (50) Pazopanib (25)	n/a	n/a	n/a	n/a	n/a	n/a			n/a	Lung (379) Lymph nodes (146) Bone (262) Liver (164) Adrenal gland (77) Soft tissue (49) Central nervous system (16)	Everolimus 233 (43.6) Temsirolsimus 178 (33.3) Sorafenib 123 (23.0)
Park (2012) [29]	Sunitinib (60) Sorafenib (16) Pazotinib (7)	n/a	0	n/a	≥ SD 66 (79.0) PD 14 (17.0) n/a 4 (5.0)	n/a	n/a			≤2 (44) ≥3 (39)	n/a	VEGF TKI 41 (49.4) mTORI: 42 (50.6)
Busch (2013) [30]	Sunitinib (20) Sorafenib (12) Bevacizumab (3) Pazopanib (1)	n/a	19 (18.4)	9.1 (6.8–11.5)	CR 1 (1.9) PR 22 (21.4) SD 42 (40.8) PD 47 (40.8)	0 (69) 1 (10) 2 (1)	n/a			1 (46) ≥3 (46)	Bone (23) Liver (23)	Sunitinib 21 (20.4) Sorafenib 39 (37.4) Everolimus 35 (34.0) Temsirolimus 5 (4.9) Other 9 (8.7)
Trask (2011) [31]	Sorafenib (62)	n/a	n/a	7.4 (6.7–11.0)	n/a	0 (21) 1 (41)	n/a			n/a	Lung (44) Node (30) Liver (20) Soft Tissue (11) Bone (8) Other (30)	Axitinib 62 (100)
Rini (2011) (a) [32]	Sunitinib (194) Cytokines (126) Bevacizumab (29) Temsirolimus (12)	n/a	n/a	n/a	n/a	0 (195) 1 (162) ≥1 (1)	100 (28.0) °	134 (37.0) °	118 (33.0) °	n/a	n/a	Axitinib 361 (100)
Rini (2011) (b) [32]	Sunitinib (195) Cytokines (125) Bevacizumab (30) Temsirolimus (12)					0 (200) 1 (160) ≥1 (0)	101 (28.0) °	130 (36.0) °	120 (33.0) °			Sorafenib 362 (100)
Zimmerman (2009) [33]	Sorafenib (22)	n/a	n/a	12.5 (n/a)	PR 7 (31.8%) SD 15 (68.2%)	n/a	10 (45.5) °	12 (54.5) °	0 (0) °	1 (3) 2 (1) ≥ 3 (18)	Lung (16) Liver (11) Lymph nodes (11) Bone (10) Brain (5)	Sunitinib 22 (100)
Di Lorenzo (2009) [34]	Interferon- alfa (5) IL-2 (4) Sunitinib (50) Sunitinib + Interferon (2)	n/a	n/a	n/a	CR 1 (1.9) PR 21 (40.4) SD 7 (13.5) PD 23 (44.2)	0 (33) 1 (15) 2 (4)	40 (76.9) °	9 (17.3) °	3 (5.78) °	1 (24) 2 (18) ≥3 (10)	Lung (38) Liver (12) Lymph nodes (12) Adrenal (5) Bone (4) Kidney (3) Soft tissue (2)	Sorafenib 52 (100)
Tamaskar (2008) [35]	Thalidomide (6) Lenalidomide (5) Volociximab (6) Bevacizumab (7) AG13736 (2) Sunitinib (5) Sorafenib (4)	n/a	n/a	n/a	n/a	n/a	n/a			n/a	Lung (21) Lymph node (18) Bone (13) Liver (11) Soft tissue (22) Brain (5)	Sunitinib and/or Sorafenib (n/a)
Motzer (2006) [36]	Interferon–apha (35) IL-2 (19) Interferon-alpha + IL-2 (9)	n/a	n/a	n/a	6%	0 (34) 1(29)	34 (54.0) °	29 (46.0) °	0 (0) °	1 (8) ≥ 2 (55)	Lung (52) Liver (10) Bone (32)	Sunitinib 63 (100)
Escudier (2004) (a) [37]	Cytokine-based (374) IL (191) Interferon (307) Both IL-2 and interferon (124)	n/a	n/a	n/a	n/a	0 (219) 1 (223) 2 (7) Unknown (2)	233 (52.0) °	218 (48.0) °	0 (0) °	1 (62) 2 (131) >2 (256) Unknown (2)	Lung (348) Liver (116)	Sorafenib 451 (100)
Escudier (2004) (b) [37]	Cytokine-based (368) IL (189) Interferon (314) Both IL-2 and interferon (135)					0 (210) 1 (236) 2 (4) Unknown (2)	228 (50.0) °	223 (50) °	0 (0) °	1 (63) 2 (129) >2 (258)	Lung (348) Liver (117)	Placebo 452 (100)

CR: Complete Response; ECOG PS: Eastern Cooperative Oncology Group Performance Status; L: Interleukin; PD: progressive disease; PD-1: programmed death-1; PFS: Progression-free survival; PR: Partial response; R: Retrospective; RCT: Randomized Controlled Trial; SD: Stable disease; EGFR-TKI: vascular endothelial growth factor receptor tyrosine kinase inhibitor; n/a: not available; ^#^: International Metastatic Renal Cell Carcinoma Database Consortium score; °: Memorial Sloan–Kettering Cancer Centre score; PFS: Progression Free Survival; n/a: not available.

**Table 3:** Clinical and pathological characteristics according to second line treatment regimens.

Characteristic	Axitinib (*n* = 532)	Cabozantinib (*n* = 365)	Nivolumab (*n* = 39)	Everolimus + Levatinib (*n* = 58)
Male:Female	400:132	277:88	29:10	42:16
Age at progression, years (mean)	64.5	63.0	63.0	59.0
**Histology of Primary Tumor, *n* (%)**				
Clear cell carcinoma	510 (95.9)	35 (9.5)	29 (74.4)	58 (100)
Non-Clear cell carcinoma	22 (4.1)	0 (0)	10 (25.6)	-
Not specified	0 (0)	330 (90.6)	0 (0)	-
**T-Stage, *n* (%)**				
T1	1 (0.2)	n/a	n/a	n/a
T2	1 (0.2)	n/a	n/a	n/a
T3	4 (0.7)	n/a	n/a	n/a
T4	59 (11.1)	n/a	n/a	n/a
Not specified	467 (87.8)	n/a	n/a	n/a
Fuhrman or WHO/ISUP Grade, *n* (%)	n/a	n/a	n/a	n/a
Prior nephrectomy, *n* (%)	156 (29.3)	268 (73.4)	34 (87.2)	50 (86.2)
**Reason for Discontinuation, *n* (%)**				
Progression	21 (3.9)	n/a	28 (71.8)	n/a
Toxicity	20 (3.7)	n/a	11 (28.2)	n/a
Not specified	491 (92.2)	n/a	0 (0)	n/a
**ECOG PS Score, *n* (%)**				
0	258 (48.5)	226 (61.9)	n/a	27 (46.5)
1	221 (41.5)	104 (28.5)	n/a	24 (41.4)
2	3 (0.6)	0 (0)	n/a	0 (0)
Not specified	50 (9.4)	35 (9.6)	n/a	7 (12.1)
**Prognostic Category, *n* (%)**				
Favorable/Good	118 (22.2)	161 (44.1)	2 (5.1)	12 (20.8)
Intermediate	207 (38.9)	156 (42.7)	23 (59.0)	23 (39.6)
Poor	136 (25.6)	48 (13.2)	14 (35.9)	23 (39.6)
Not specified	71 (13.3)	0 (0)	0 (0)	-
**Metastatic sites, *n* (%)**				
1	25 (4.7)	6 (1.6)	21 (53.8)	18 (31.0)
≥2	22 (4.1)	14 (3.8)	18 (46.2)	33 (56.9)
Not specified	485 (91.2)	345 (94.6)	0 (0)	7 (12.1)
**Involved Metastatic Sites, *n* (%)**				
Lung	73 (13.7)	21 (5.7)	n/a	33 (56.9)
Liver	24 (4.5)	9 (2.5)	n/a	11 (18.9)
Lymph node	39 (7.3)	11 (3.0)	n/a	25 (43.1)
Bone	16 (3.0)	8 (2.2)	n/a	15 (25.9)
Other	53 (9.9)	15 (4.1)	n/a	4 (6.9)
Not specified	408 (77.7)	330 (90.4)	n/a	0 (0)

Percentage are calculated on the total number of patients treated with the specific second-line regimen.
